# Threshold Ferritin Concentrations Reflecting Early Iron Deficiency Based on Hepcidin and Soluble Transferrin Receptor Serum Levels in Patients with Absolute Iron Deficiency

**DOI:** 10.3390/nu14224739

**Published:** 2022-11-10

**Authors:** Laura Tarancon-Diez, Miguel Genebat, Manuela Roman-Enry, Elena Vázquez-Alejo, Maria de la Sierra Espinar-Buitrago, Manuel Leal, Mª Ángeles Muñoz-Fernandez

**Affiliations:** 1 Immunology Section, Laboratorio Inmuno-Biología Molecular (LIBM), Hospital General Universitario Gregorio Marañón, Instituto de Investigación Sanitaria Gregorio Marañón (IiSGM), 28009 Madrid, Spain; 2 Internal Medicine Department, Hospital Fátima, 41012 Sevilla, Spain; 3 AnAliza laboratorio, Hospital Jerez Puerta del Sur, 11408 Cádiz, Spain; 4 Internal Medicine Department, Hospital Viamed Santa Ángela de la Cruz, 41014 Sevilla, Spain

**Keywords:** ferritin, iron deficiency, hepcidin, soluble transferrin receptor

## Abstract

(1) Background: The serum ferritin cut-off to define absolute iron deficiency is not well-established. The aim of the present study was to determine a clinically relevant ferritin threshold by using early serum biomarkers of iron deficiency such as hepcidin and the soluble transferrin receptor; (2) Methods: Two hundred and twenty-eight asymptomatic subjects attending a hospital as outpatients between 1st April 2020 and 27th February 2022 were selected. Iron metabolism parameters as part of the blood analysis were requested by their doctor and included in the study. Then, they were classified into groups according to their ferritin levels and iron-related biomarkers in serum were determined, quantified, and compared between ferritin score groups and anemic subjects. (3) Results: Serum ferritin levels below 50 ng/mL establish the point from which the serum biomarker, the soluble transferrin receptor to hepcidin ratio (sTfR/Hep ratio), begins to correlate significantly with ferritin levels. (4) Conclusion: Ferritin levels ≤ 50 ng/mL are indicative of early iron deficiency; hence, this should be considered as a clinically relevant cut-off for iron deficiency.

## 1. Introduction

Iron deficiency is one the main causes of anemia, and it may be caused by a wide variety of different clinical factors, such as an insufficient iron diet intake, malabsorption, or chronic bleeding [1]. Some of these causes of iron deficiency may be life-threatening, such as colorectal cancer. Hence, an accurate early detection of ferropenia is required in order to make a diagnosis and to start specific treatment if possible, avoiding future blood or hemoderivative transfusions.

Apart from the diagnosis of underlying causes, iron deficiency must be treated with iron supplementation in order to improve related symptoms, independently of the cause of ferropenia and the presence or absence of anemia [2]. Iron is not only necessary for erythropoiesis; it is necessary for the cellular metabolism of all organs and systems, especially those with a high cell turnover, including immune cells [3,4]. Thus, in recent years, the role of iron in the immune response to infections [5] and in vaccine responses [6,7] has acquired great relevance.

It remains unclear when a correct iron deficiency diagnosis should be made. The gold standard to establish a diagnosis has been the invasive method of bone marrow aspiration to show iron absence or decline. However, both the heterogeneous distribution of iron stores in the bone marrow along with logistical problems regarding aspiration make this test not appropriate for routine examinations and contribute to the urgent search for alternative methods for iron deficiency diagnosis. Thus, currently, the ferritin plasma level is the preferred surrogate marker to define iron deficiency, although the exact threshold has not been established, ranging from less than 15 ng/mL to 100 ng/mL [8,9,10,11]. Experts markedly recommend the use of higher cut-offs to avoid false-negative tests that would leave iron deficiency untreated. In the case of Galleti et al., they recently communicated a ferritin cut-off of <50 mg/L as an indicator of incipient iron deficiency in young women [10].

Hepcidin is an iron metabolism regulatory hormone, mainly produced in the liver, closely related with ferritin levels; the soluble transferrin receptor (sTfR) is a cleaved extracellular portion of the cellular receptor. Both biomarkers have been used as additional plasma markers of iron status but only for research purposes and not in routine clinical settings [12]. Hence, the objective of the present article is to establish a clinically relevant cut-off for ferritin levels to define iron deficiency, based on both plasma biomarkers (hepcidin and sTfR) as predictors of iron deficiency.

## 2. Materials and Methods

### 2.1. Patients

This retrospective and observational study was performed on subjects who consecutively visited the Hospital Viamed Santa Ángela de la Cruz in Sevilla, Spain, as outpatients between 1 April 2020 and 27 February 2022, and whose doctor requested an iron metabolism study as part of routine clinical management in their blood analysis.

From 339 candidates with these characteristics, 228 subjects were included, since they fulfilled the inclusion criteria for the present study, which were as follows: (i) age between 18 and 65 years old (because of the known effect of inflammaging above 65 years of age); (ii) body mass index (BMI) < 40 kg/m^2^ (in order to avoid obesity or overweight related inflammation and iron absorption defect); (iii) clinically asymptomatic when the blood analysis was performed; (iv) no hospitalization due to any cause in the prior month, no chronic renal disease, no decompensated heart failure, no acute or chronic infection (HIV or hepatitis C virus), no autoimmune disease or onco-hematological disease; (v) no recent use of iron supplements or nutritional supplements potentially containing iron; (vi) no blood transfusion, immunomodulatory treatment or chemotherapy in the prior three months; and (vii) non-pregnant and non-breastfeeding women.

The present study was approved by the local Ethical Committee from Virgen Macarena and Virgen del Rocio University Hospitals (PEIBA Acta CEI_03/2022). The study was conducted in accordance with the Declaration of Helsinki.

### 2.2. Blood Analyses and Iron Absorption Determination

Subjects’ height and weight for BMI were measured using standardized methods. For hematological analysis, an overnight fasting venous blood sample in the morning was collected. Traditional iron-related biomarkers were determined in plasma samples by means of standardized techniques at the Laboratory Service of Hospital Viamed Santa Ángela. Briefly, total iron (sideremia), transferrin, and soluble transferrin receptor (sTfR) were measured by photometry and ferritin by means of the particle enhanced immunoturbidimetric assay in a Hitachi Cobas C702 modular analyzer (Roche Diagnostics, Rotkreuz, Switzerland). The transferrin saturation index (TfSI) was estimated as total plasma iron (µg/dL) × 100)/transferrin (mg/dL) × 1.27. For all samples, plasma hepcidin was determined by ELISA (Hepcidin25 HS ELISA, DRG).

### 2.3. Statistical Analysis

Continuous variables were expressed as median and interquartile range [IQR], while categorical ones were expressed as the number of subjects and percentage (%). Differences between categorical and continuous values were determined using the chi-square test and the Mann–Whitney U-test, respectively; Spearman’s test was applied to analyze the correlation between ferritin levels and different biomarkers; *p* values < 0.05 were considered statistically significant. The Statistical Package for the Social Sciences software (SPSS 20.0, Chicago, IL, USA) and GraphPad Prism 9.0 (GraphPad Software, Inc., San Diego, CA, USA) were used for the statistical analysis and graphs generation.

## 3. Results

**Figure 1:**
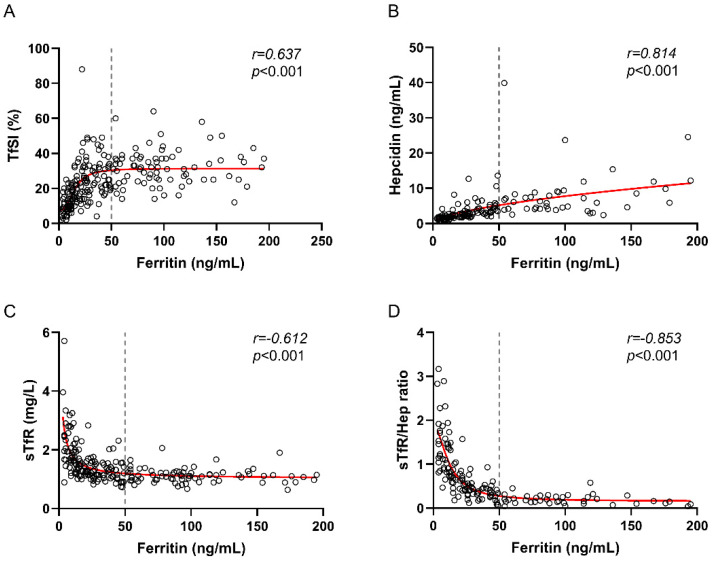
Correlations between early iron deficiency serum biomarkers and serum ferritin. Serum ferritin correlations with TfSI (**A**), Hepcidin (**B**), sTfR (**C**) and sTfR/Hep ratio (**D**) in all participants. Gray dashed line is the ferritin cut-off of 50 ng/mL. Abbreviations: TfSI, transferrin saturation index; sTfR, soluble transferrin receptor; Hep, hepcidin. Spearman’s correlation test was used.

General characteristics and soluble iron-related biomarkers of the 228 included participants are shown in Table 1. It should be noted that 86% were women and had a median age of 44 years (IQR: 35–51) and a median BMI of 24.3 (IQR: 21.5–28). Iron-related biomarkers included hemoglobin (HB), mean corpuscular hemoglobin (MCH), mean corpuscular hemoglobin concentration (MCHC), mean corpuscular volume (MCV), transferrin, TfSI, sideremia, ferritin, sTfR, hepcidin (Hep) and sTfR to hepcidin ratio (sTfR/Hep ratio). A total of 24 patients (10.5%) had anemia (defined as hemoglobin levels below 13 gr/L in men and below 12 gr/L in women) and they showed a median of serum ferritin of 8 [IQR: 5–14.25]. As shown in Figure 1, serum ferritin strongly and directly correlated with TfSI and Hep, and inversely with sTfR and sTfR/Hep ratio (*p* < 0.001 for all comparisons) (Figure 1A–D).

**Figure 2:**
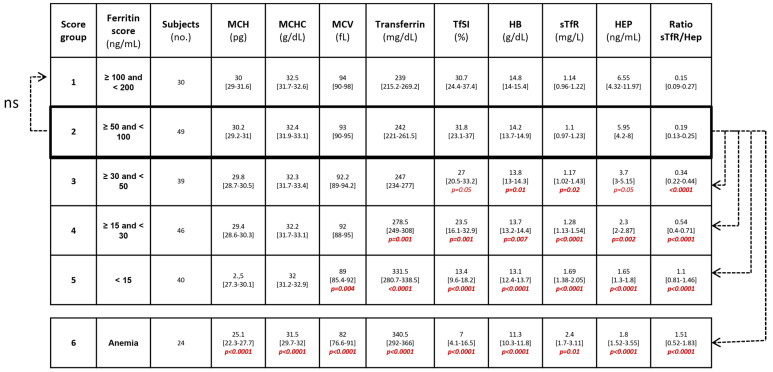
Comparisons of iron biomarkers between different ferritin score groups and anemic subjects. All score groups were compared with group 2, which was considered as the reference group. Abbreviations: HCH, mean corpuscular hemoglobin; MCHC, mean corpuscular hemoglobin concentration; MCV, mean corpuscular volume; TfSI, transferrin saturation index; HB, hemoglobin; sTfR, soluble transferrin receptor; HEP, hepcidin. Continuous variables are expressed as the medians and interquartile ranges (IQR). Categorical variables are expressed as numbers and percentages. Differences between variables and groups were determined by using the Mann–Whitney U-test. ns: not statistically significant.

Excluding anemic subjects, all participants were then categorized into groups according to serum ferritin levels to establish the limit from which other biomarkers start to predict iron deficiency. The ferritin score groups were defined as follows: score 1: ferritin levels ≥100 and <200 ng/mL; score 2: ferritin levels ≥50 and <100 ng/mL; score 3: ferritin levels ≥30 and <50 ng/mL; score 4: ferritin levels ≥15 and <30 ng/mL and score 5: ferritin levels <15 ng/mL. Subjects with anemia were considered to have a score of 6. Comparisons between groups showed statistical differences among the variables comparing the score 2 group with scores 3, 4, 5 and 6 (Figure 2). Hence, the ferritin levels from the score 2 group were the highest cut-off limit with significantly different levels of sTfR/Hep ratio (*p* < 0.001), sTfR (*p* < 0.02) and HB (*p* < 0.01). Thus, this limit could establish the point from which the other serum biomarkers such as the sTfR/Hep ratio begins to correlate significantly with ferritin levels and could be considered as a cut-off for iron deficiency. According to the cut-off of 50 ng/mL of ferritin that we established, 149 (65%) of all participants presented iron deficiency, and of them, 24 (16%) had developed anemia, reflecting the clinical relevance of iron deficiency independently of the presence of anemia.

**Figure 3:**
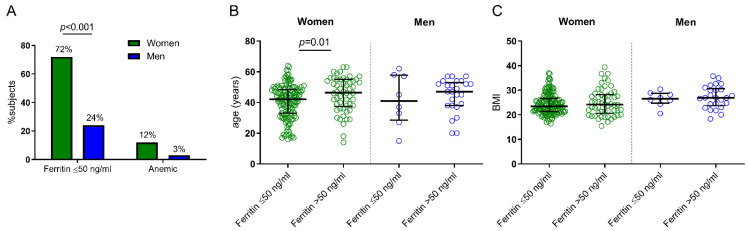
Iron deficiency and anemia frequency and differences in age and BMI depending on ferritin cut-off. Frequency of subjects with serum ferritin levels ≤ 50 ng/mL and with anemia in women and men (**A**); and differences in age (**B**) and BMI (**C**) depending on serum ferritin cut-off of 50 ng/mL in women and men. BMI: body mass index. Differences between categorical and continuous values were determined using the chi-square test and Mann–Whitney U-test, respectively. Only statistically significant differences with *p*-value < 0.05 are shown.

**Figure 4:**
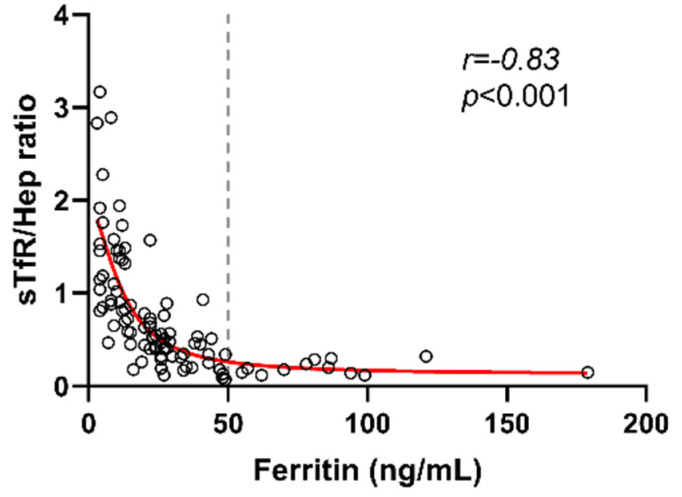
Correlation between sTfR to Hepcidin ratio and ferritin levels in women younger than 50 years old (data available for 96 women). Gray dashed line is the ferritin cut-off of 50 ng/mL. Abbreviations: sTfR, soluble transferrin receptor; Hep, hepcidin. Spearman’s correlation test was used.

Trying to evaluate a sex-dependent cut-off, a similar ferritin score group categorization was performed only in women (n = 195), and similar results were obtained comparing scores groups and anemic women (Supplementary Figure S1). The low frequency of men (n = 33) was not enough to perform the analysis segregating them into six score groups. However, it should be noted that, based on our cut-off of ≤50 ng/mL, the frequency of iron deficiency in men was significantly lower compared with women (24% vs. 72%), while no significant differences were observed in the prevalence of anemia (3% in men vs. 12% in women) (Figure 3A). Based on the ferritin cut-off of 50 ng/mL, women with serum concentration below that limit had a significantly lower age compared with women with high ferritin levels, while no differences were observed in men (Figure 3B). Regarding the BMI, no differences were observed between groups (Figure 3C). Finally, the analysis including only premenopausal women younger than 50 years old (n = 143) also showed a strong and inversed correlation between ferritin levels and sTfR/Hep ratio (*p* < 0.001; r = −0.83) (Figure 4), and comparing ferritin score groups and anemic premenopausal women, ferritin levels from the score 2 group were also the highest cut-off limit with significantly different levels of sTfR/Hep ratio (*p* = 0.03) (Supplementary Figure S2).

## 4. Discussion

The results presented herein suggest that ferritin levels ≤ 50 ng/mL could be indicative of early iron deficiency, mainly based on the behavior of the sTfR to hepcidin ratio, showing that this threshold could be considered as the clinically relevant cut-off for absolute ferropenia in order to perform an early diagnosis of potential causes and to start specific treatment.

The World Health Organization’s (WHO) recommendations for biomarkers for iron deficiency suggest a cut-off below 15 ng/mL in a general healthy population [8]. Interestingly, the findings in this study confirm recently published results by Galetti et al., where they also set the cut-off as below 50 ng/mL, calculated by using the iron isotopic label [10]. This study reached the same reference cut-off but through a novel experimental approach of using serum biomarkers that could be implemented in general clinical practice and replace isotopic techniques. The similar behavior of this isotopic biomarker and the sTfR to hepcidin ratio described here when ferritin levels remain below 50 ng/mL should be noted. In line with the present results, a close association between iron deposits in bone marrow aspirates and serum sTfR concentration was recently reported, albeit in a different clinical setting [13].

As far as we know, this is the first study in which the sTfR to hepcidin ratio has been used. Among multiple iron metabolism biomarkers assessed, the sTfR to hepcidin ratio stands out in this study as the variable that most significantly correlated with ferritin levels. There has been much interest in the clinical significance of sTfR and hepcidin individually, as they are known as markers of iron deficiency, anemia, chronic diseases and, more recently, both have been related to all-cause mortality regardless of anemia and iron storage status [13,14,15,16,17,18]. Thus, several forms of anemia may benefit from therapies based on hepcidin-lowering agents or antagonists [19,20].

This study also found a sex-dependent ferropenia, with outstanding prevalence in women, in combination with a clear association with age on female group (Figure 3B). Women very often have a specific behavior regarding iron metabolism due to excessive menstruation bleeding and elevated iron requirements during pregnancy [21] and, as was expected, in the present research, the highest ferritin levels were found in older women. This is in agreement with previous observations in which women at reproductive age were at increased risk of anemia and iron deficiency and also with previous observations in which the median serum ferritin concentrations in European women at reproductive age were estimated at 26–38 μg/L [22]. However, the sub-analysis carried out only on women below 50 years old showed similar results.

Iron deficiency is particularly frequent in obese patients due to increased circulating levels of acute-phase reactant hepcidin and adiposity-associated inflammation, which result in a decrease in Fe absorption and immobilization of iron trapped in deposits [23,24]. Zimmermann et al. reported that a high BMI was associated with decreased Fe absorption [25]. However, the present study did not show a BMI-dependent iron deficiency either in women or in men, probably due to the low frequency of participants (15%) within the range considered obese (BMI of 30 or higher).

Establishing a clinically relevant threshold for absolute ferropenia is exceptionally important, since no consensus has been achieved up to now. In addition, it is considered that around 30% of the global population will develop ferropenia during their live, which is the main cause of anemia. Based on the novel established threshold of soluble ferritin at ≤50 ng/mL, the prevalence of iron deficiency could be as high as 65%. This considerable gap with what has been described so far and current estimations suggests a possible underdiagnosis in ferropenia in the general population, and thus the consequent underestimation of possible underlying iron deficiency-related pathologies. Hence, it is mandatory to establish the level of ferropenia reflecting early iron deficiency, even without anemia. According to our results, we consider that iron metabolism determinations should be ordered in every routine blood analysis to promptly detect iron deficiency requiring further investigation and treatment. However, despite these findings, hepcidin and sTfR are not yet included in the blood analysis routinely, since these serum biomarkers have only been used to establish the ferritin cut-off.

In addition to its clear role in iron deficiency, and increased level of ferritin has recently received increased interest as an acute phase reactant and a simple marker of inflammation [26]. In the present study, the lack of data on inflammation-related cytokines or biomarkers prevents us from identifying ferritin and of the other iron-related biomarkers as surrogates for systemic inflammation. Apart from that, this study has other limitations. First, the huge range of ages may limit the interpretation of results. Secondly, the menstrual cycle phase was not taken into consideration regarding when blood was taken from the women. In addition, our proposed ferritin threshold of 50 ng/mL should only be applied to young women, and may not be applicable to men, who were scarcely represented in the present study, and to functional iron deficiency in inflammatory pathologies.

## 5. Conclusions

Our results suggest that a ferritin level < 50 ng/mL is the clinical threshold to redefine and consider ferropenia, in order to achieve an early diagnosis and initiate treatment for iron deficiency.

## Figures and Tables

**Table 1:** Baseline characteristics of the patients.

Number of Subjects	228
Female sex (%)	195 (86)
Age (years)	44 (35–51)
Body mass index (Kg/m^2^)	24.3 (21.5–28)
Body mass index ≥ 30 (%)	34 (15)
Hemoglobin (g/dL)	13.8 (12.8–14.4)
Mean corpuscle volume (fL)	91.4 (88–94.8)
Mean corpuscle hemoglobin concentration (pg)	29.5 (28.3–30.5)
Subjects with anemia (%)	24 (11)
Transferrin level (mg/dL)	268.5 (238.3–306.3)
Saturation transferrin index	23.1 (14.4–32.6)
Sideremia (µg/dL)	77 (54–105)
Ferritin (ng/mL)	34 (14–74)
Subjects with ferritin < 50 (ng/mL)	149 (65)
Soluble transferrin receptor (mg/L) ^a^	1.31 (1.28–1.66)
Hepcidin (ng/mL) ^b^	2.9 (1.8–5.2)

^a,b^ Data available for 216 and 153 participants, respectively. Continuous variables are expressed as the medians and interquartile ranges (IQR). Categorical variables are expressed as numbers and percentages.

## Data Availability

The data presented in this study are available on request from the corresponding author.

## Supplementary Materials

The following supporting information can be downloaded at: https://www.mdpi.com/article/10.3390/nu14224739/s1, Figure S1: Comparisosn of iron biomarkers between different ferritin score groups and anemic women (n = 195); Figure S2: Comparisosn of iron biomarkers between different ferritin score groups and anemic women younger than 50 years old (n = 143).
