# Supplementary material for: Threshold Ferritin Concentrations Reflecting Early Iron Deficiency Based on Hepcidin and Soluble Transferrin Receptor Serum Levels in Patients with Absolute Iron Deficiency
Source: Nutrients. 2022 Nov 10;14(22):4739. doi: 10.3390/nu14224739 (PMC9692751; doi:10.3390/nu14224739)
Supplement: Supplementary file 1 [file nutrients-14-04739-s001.zip › nutrients-2010684-supplementary.pdf]

Supplementary material:

ns

Score group	Ferritin score (ng/mL)	Subjects (no.)	MCH (pg)	MCHC (g/dL)	MCV (fL)	Transferrin (mg/dL)	TfSI (%)	HB (g/dL)	sTfR (mg/L)	HEP (ng/mL)	Ratio sTfR/Hep
1	≥ 100 and < 200	13	29.9 [29-32]	31.9 [31.5-32.3]	94 [89-99]	230 [213-262]	28 [24.8-35.9]	14 [13-14.7]	1.09 [0.9-1.2]	5.9 [2.9-17.8]	0.15 [0.07-0.45]
2	≥ 50 and < 100	41	30.2 [29.2-30.9]	32.3 [31.7-33.1]	93 [90.5-95]	242 [222-262]	32 [23.4-37.2]	14 [13.6-14.5]	1.07 [0.96-1.23]	6 [4.2-8.1]	0.19 [0.13-0.24]
3	≥ 30 and < 50	35	30 [29-30.6]	32.3 [31.8-33.4]	93 [89.8-94.2]	247 [234-278]	27.8 [21-33.5]	13.7 [12.8-14.2] *p=0.02*	1.14 [1-1.45]	3.7 [3-5.3] *p=0.007*	0.33 [0.21-0.44] *p=0.001*
4	≥ 15 and < 30	44	29.4 [28.7-30.57]	32.2 [31.7-33.2]	92 [88-95]	278 [249-307] *<0.0001*	23.2 [16-32.2] *p=0.001*	13.7 [13.12-14.3]	1.27 [1.13-1.55] *<0.0001*	2.3 [1.97-2.95] *<0.0001*	0.52 [0.4-0.74] *<0.0001*
5	< 15	39	28.5 [27.3-30.1] *<0.0001*	32.1 [31.3-32.9]	89.1 [85.2-92] *<0.0001*	312 [280-340] *<0.0001*	13.8 [10-18.5] *<0.0001*	13.1 [12.4-13.8] *<0.0001*	1.66 [1.36-2.05] *<0.0001*	1.6 [1.3-1.8] *<0.0001*	1.11 [0.81-1.47] *<0.0001*
6	Anaemia	23	25.5 [22.5-27.8] *<0.0001*	31.5 [29.7-32]	83 [77-91] *<0.0001*	336 [287-362] *<0.0001*	7.16 [4.21-18] *<0.0001*	11.4 [10.3-11.8] *<0.0001*	2.33 [1.7-3.18] *<0.0001*	1.8 [1.6-3.9] *<0.0001*	1.45 [0.47-1.75] *<0.0001*

Figure S1*: Comparisosn of iron biomarkers between different ferritin score groups and anemic women (n=195). All score groups were compared with group 2 that was considered the reference group. Abbreviations: HCH, mean corpuscular haemoglobin; MCHC, mean corpuscular haemoglobin concentration, MCHC; MCV, mean corpuscular volume; TfSI, transferrin saturation index; HB, hemoglobin; sTfR, soluble transferrin receptor; HEP, hepcidin. Continuous variables are expressed as the medians and interquartile ranges [IQR]. Categorical variables are expressed as numbers and percentages. Differences between variables and groups were determined by using Mann Whitney U-test.*

Score group	Ferritin score (ng/mL)	Subjects (no.)	MCH (pg)	MCHC (g/dL)	MCV (fL)	Transferrin (mg/dL)	TfSI (%)	HB (g/dL)	sTfR (mg/L)	HEP (ng/mL)	Ratio sTfR/Hep
**1**	**≥ 100 and < 200**	6	29.8 [28.9-30.7]	32 [31.7-32.8]	94 [87-95.5]	220 [203.5-305]	27.5 [24-35]	14.1 [13.7-15]	0.92 [0.82-1.17]	4.45 [3-5.9]	0.23 [0.15-0.32]
**2**	**≥ 50 and < 100**	25	30 [29.2-31]	32.3 [31.7-33.1]	93 [90-94.9]	242 [222-258.5]	31 [23.8-37.3]	14 [13.5-14.35]	1.1 [0.98-1.23]	5.15 [4.2-8]	0.19 [0.13-0.24]
**3**	**≥ 30 and < 50**	25	30.4 [29-31.2]	32.3 [31.9-33.5]	92 [89.4-94.3]	256 [233-277]	30.9 [20.5-38.5]	13.3 [12.7-14.2]	1.09 [0.93-1.32]	4.2 [3-6.2]	0.31 [0.18-0.45] ***p=0.03***
**4**	**≥ 15 and < 30**	38	29.6 [28.7-30.7]	32.2 [31.7-33.3]	92 [88-95]	272 [246-295] ***p=0.002***	23.8 [17-33] ***p=0.03***	13.7 [13.2-14.3]	1.27 [1.12-1.48] ***p=0.006***	2.5 [2-3.1] ***<0.0001***	0.48 [0.39-0.62] ***<0.0001***
**5**	**< 15**	32	28.5 [27.3-30.2] ***p=0.006***	32.1 [31.3-33]	89 [85.2-91.9] ***p=0.002***	310 [273-339] ***<0.0001***	13.4 [9.4-18] ***<0.0001***	13 [12.4-13.6] ***<0.0001***	1.66 [1.35-2.02] ***<0.0001***	1.6 [1.3-1.8] ***<0.0001***	1.12 [0.82-1.47] ***<0.0001***
**6**	**Anaemia**	17	25.5 [23.1-27.6] ***<0.0001***	30.4 [29.5-32] ***<0.0001***	83 [77.2-90] ***<0.0001***	345 [280-369] ***<0.0001***	7.16 [4-19] ***<0.0001***	11.4 [10-11.8] ***<0.0001***	2.64 [1.72-3.27] ***<0.0001***	1.8 [1.4-3.7] ***<0.0001***	1.5 [0.5-2.56] ***<0.0001***

ns

Figure S2: *Comparisosn of iron biomarkers between different ferritin score groups and anemic women younger than 50 years old (n=143). All score groups were compared with group 2 that was considered the reference group. Abbreviations: HCH, mean corpuscular haemoglobin; MCHC, mean corpuscular haemoglobin concentration, MCHC; MCV, mean corpuscular volume; TfSI, transferrin saturation index; HB, hemoglobin; sTfR, soluble transferrin receptor; HEP, hepcidin. Continuous variables are expressed as the medians and interquartile ranges [IQR]. Categorical variables are expressed as numbers and percentages. Differences between variables and groups were determined by using Mann Whitney U-test.*
